# Large-Scale Integrative Analysis of Soybean Transcriptome Using an Unsupervised Autoencoder Model

**DOI:** 10.3389/fpls.2022.831204

**Published:** 2022-03-03

**Authors:** Lingtao Su, Chunhui Xu, Shuai Zeng, Li Su, Trupti Joshi, Gary Stacey, Dong Xu

**Affiliations:** ^1^Department of Electrical Engineering and Computer Science and Christopher S. Bond Life Sciences Center, University of Missouri, Columbia, MO, United States; ^2^Institute for Data Science and Informatics, Christopher S. Bond Life Sciences Center, University of Missouri, Columbia, MO, United States; ^3^Department of Health Management and Informatics and Christopher S. Bond Life Sciences Center, University of Missouri, Columbia, MO, United States; ^4^Division of Plant Sciences and Technology and Biochemistry Christopher S. Bond Life Sciences Center, University of Missouri, Columbia, MO, United States

**Keywords:** soybean, transcriptome analysis, deep learning, autoencoder, tissue-specific gene, gene regulatory network, functional module

## Abstract

Plant tissues are distinguished by their gene expression patterns, which can help identify tissue-specific highly expressed genes and their differential functional modules. For this purpose, large-scale soybean transcriptome samples were collected and processed starting from raw sequencing reads in a uniform analysis pipeline. To address the gene expression heterogeneity in different tissues, we utilized an adversarial deconfounding autoencoder (AD-AE) model to map gene expressions into a latent space and adapted a standard unsupervised autoencoder (AE) model to help effectively extract meaningful biological signals from the noisy data. As a result, four groups of 1,743, 914, 2,107, and 1,451 genes were found highly expressed specifically in leaf, root, seed and nodule tissues, respectively. To obtain key transcription factors (TFs), hub genes and their functional modules in each tissue, we constructed tissue-specific gene regulatory networks (GRNs), and differential correlation networks by using corrected and compressed gene expression data. We validated our results from the literature and gene enrichment analysis, which confirmed many identified tissue-specific genes. Our study represents the largest gene expression analysis in soybean tissues to date. It provides valuable targets for tissue-specific research and helps uncover broader biological patterns. Code is publicly available with open source at https://github.com/LingtaoSu/SoyMeta.

## Introduction

The soybean is a valuable source of oil and protein for humans and livestock; it is also very important for soil fertility, given the symbiotic interaction with nitrogen-fixing rhizobia. The development of high-throughput gene expression quantification technologies, initially dominated by microarray platforms and later by RNA-Seq technologies, has contributed to the substantial rise in soybean transcriptome studies. The related data are well presented in several public data repositories, such as the SoyKB (Joshi et al., 2012, 2014, 2017), SoyBase (Grant et al., 2010; Brown et al., 2021) and the most recently published Soybean Expression Atlas (Machado et al., 2020), covering thousands of gene expression data sets from various tissues, developmental stages and conditions. Typically, several related experiments studying the same tissue are available, providing a rich set of materials for integrative analysis via data pooling and mining. The increasing sample size also enhances the statistical power to obtain a more precise and robust estimate of molecular markers and reduces the noise effects and individual study biases.

Researchers have become increasingly interested in integrating their own data with publicly available data sets to achieve more accurate results and deeper biological understanding. For example, through a large-scale transcriptome meta-analysis, several hub genes involved in soybean oil accumulation processes were revealed in Qi et al. (2018), and a large number of differentially expressed genes (DEGs) related to soybean symbiotic nitrogen fixation were also identified (Yuan et al., 2017). In addition, some similar studies are available (Liu et al., 2015; Huang et al., 2018; Wang J. et al., 2019; Yi et al., 2019). However, most of these investigations explored only a few conditions or developmental stages. Such *ad hoc* approaches can overlook a myriad of interesting transcriptional patterns, which could otherwise be unraveled by integrative methods using a more comprehensive set of samples. Inspired by this, a global co-expression network analysis of 1,072 soybean microarray samples was conducted (Wu et al., 2019), which revealed a gene module that is likely involved in the evolution of nodulation in plants. Kim et al. (2017) constructed the SoyNet database using 734 microarrays and 290 RNA-seq samples. Moreover, by systematically analyzing 1,270 microarray samples generated with Affymetrix gene chips, a nodulation-related co-expression module was uncovered (Wu et al., 2019). More recently, researchers (Sun et al., 2020) identified key regulators and hub genes in each tissue by analyzing a genome-wide transcriptome dataset from eight tissues at three different seed development stages. To elucidate the dynamics of transcriptional regulation across the broad range of samples, tissues, and cultivars, 1,298 publicly available soybean transcriptome samples were collected and analyzed by Machado et al. (2020).

Properly integrating large-scale data sets can help increase statistical power, but expression profiles inherently contain variations introduced by noise, batch effects and conditions unrelated to the biological hypotheses. Although many batch effect adjustment methods have been proposed (Benito et al., 2004; Johnson et al., 2007; Sims et al., 2008; Luo et al., 2010; Xia et al., 2013), they typically cannot handle large-scale data integration (Haibe-Kains et al., 2013). Therefore, the integration of microarray and RNA-seq data sets continues to be a challenging problem (Lazar et al., 2013). However, the emergence of deep learning techniques provides a new perspective and opportunity to solve this problem. The unsupervised learning model can extract patterns from diverse and noisy data without assuming any statistical properties of the data, which makes it well suited for gene expression analysis (Du et al., 2019; Dincer et al., 2020; Li et al., 2020). For example, the adversarial deconfounding autoencoder (AD-AE) model (Dincer et al., 2020) can generate biologically informative expression embeddings that are both robust to confounders and generalizable. The AD-AE model uses an autoencoder network to capture the true signal and a complete adversary network to remove confounder variables for a noise-free and confounder-free representation. Considering the widespread noise in soybean gene expression datasets (Araujo et al., 2017; Cortijo et al., 2019), in this study, we adapted the AD-AE model to analyze collected soybean datasets, considering not only different data sources but also different sequencing platforms.

As more and more tissue-specific gene expression data become available for soybeans (Libault et al., 2010), another important aspect in the large-scale integrative analysis is detecting tissue-specific genes and constructing tissue-specific gene regulatory networks (GRNs). Several computational methods were developed to measure the tissue specificity of gene expression, such as the EE (Yu et al., 2006) in the database TiGER (Liu et al., 2008), the SPM used in the database TiSGeD (Xiao et al., 2010), and the Gini coefficient (Ceriani and Verme, 2012). However, all these methods need to calculate the mean expression value and the expression maximum value for each gene in each tissue as a global measure of the gene’s specificity. One disadvantage of such methods, especially when there are a large number of samples for each tissue, is that confounding variation and noise may hinder learning biologically meaningful representations. Autoencoder-based data compression is preferred in this case to efficiently extract the true signal from high dimensional data and to learn latent representations corresponding to biological information of interest (Gupta et al., 2015; Lin et al., 2017; Xie et al., 2017; Ding et al., 2018). Therefore, we propose the use of an autoencoder model for detecting tissue-specific highly expressed genes. As for GRN construction, GENIE3 is a widely used tool (Marbach et al., 2012) and has been successfully applied in constructing the Arabidopsis (Ezer et al., 2017) and maize (Walley et al., 2016) GRNs. However, GENIE3 is very time consuming especially when the gene expression vector is in high dimension.

Furthermore, in GRN construction, without dimension reduction, confounder-based variations often mask true signals of biologically meaningful regulations (Yi et al., 2018; Kinalis et al., 2019). To address these issues, we applied an autoencoder to compress our sample representations in each tissue into a much lower dimension, i.e., to learn a latent space that maps M samples to D dimension (M ≫ D) such that the biological signals presented in the original expression space can be preserved in the D-dimensional space. Then, we constructed and compared the differential regulatory network for each tissue using the embedded expression matrix produced by the autoencoder model. Moreover, key transaction factors (TFs) were identified for each tissue. We also predicted the functional modules in each differential network by performing clustering analyses.

**FIGURE 1:**
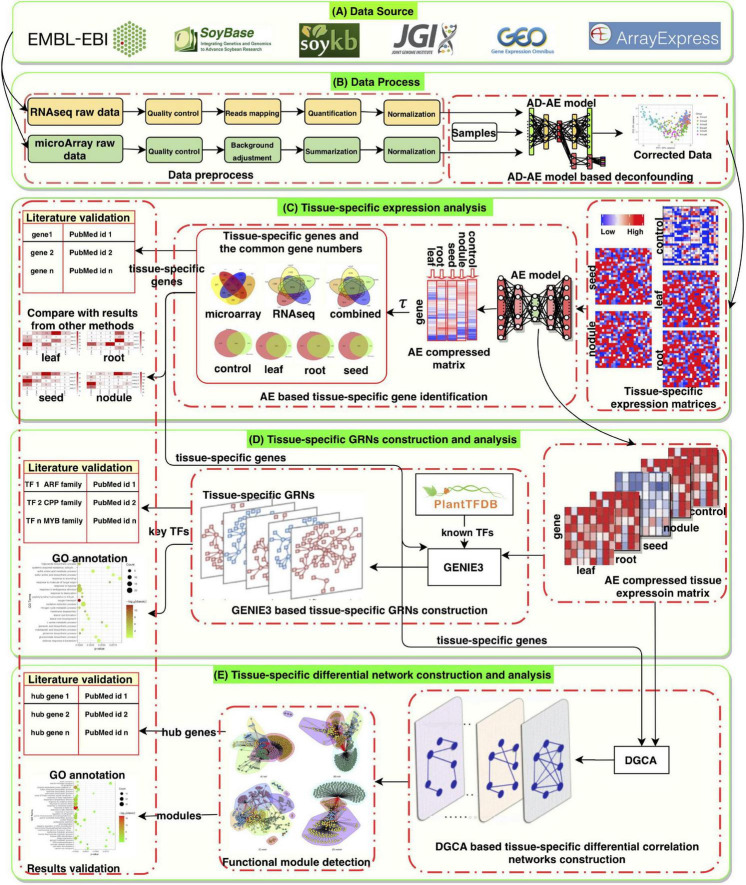
The overall framework and workflow.

In this study, after processing, 5,422 high-quality samples were left for analysis (either RNA-seq or microarray data) and were manually separated into case and control (“baseline”) groups. Each group contains gene expression profiles of many different tissues and development stages from a wide range of studies. The control expression was obtained under normal, untreated conditions. Each sample was manually curated, and both microarray and RNA expression levels were mapped and normalized based on original raw sequencing reads. With the data sets and the autoencoder model, we identified highly expressed genes in the soybean leaf, root, seed, and nodule. In combination with GENIE3 and the autoencoder model, tissue-specific GRNs were constructed, and hub TFs were identified. After comparing our newly constructed GRNs with the corresponding control network, a differential correlation network was constructed. This network provided us the opportunity to identify new genes and interactions with significant changes. We also clustered the network modules and provided their functional annotations. Figure 1 shows the overall framework and our workflow, which includes five parts: (A) the data source, (B) the data process, (C) tissue-specific expression analysis, (D) tissue-specific GRN construction and analysis, and (E) tissue-specific differential network construction and analysis. To the best of our knowledge, this is the largest analysis of gene expressions in soybean tissues to date. Our results can provide more accurate targets for future tissue-specific studies and help uncover broader biological patterns.

## Materials and Methods

### Data Collection, Processing, and Normalization

Selecting and pre-processing suitable microarray and RNA-seq datasets, are key issues and steps in conducting large-scale integrating analysis. After a systematic review of soybean-related microarray studies in the literature, we found a large number of samples sequenced by the Affymetrix GPL4592 (Affymetrix Glycine max Genome Array) platform. For easy data integration and normalization, only data generated from the GPL4592 platform were used for microarray datasets in this study. The RNA-Seq samples were mainly downloaded from the NCBI SRA (Leinonen et al., 2011) and the ArrayExpress database (Athar et al., 2019) together with some in-house data. Each dataset was manually checked using the following three criteria: (i) methods for the sequencing experiments, (ii) available raw data (FASTQ or SRA formats), and (iii) detailed sample information to determine whether it was included in the analysis or not. Reads obtained from the same biological sample were combined in a single FASTQ file (or in two files, for paired-end data).

The analysis pipeline is shown in Figure 1B, where the microarray and RNA-seq data are processed separately with different tools for quality check, raw data processing and normalization. In detail, for RNA-seq data, we used TrimGalore-0.6.5^1^ to trim sequence adapters from the raw reads FASTQ files. The trimmed FASTQ files were then prepared for quality control with FastQC-0.11.8 (Wingett and Andrews, 2018), which provides a quick view on the quality of the raw sequence reads from multiple analyses, ranging from the sequence quality and GC content to library complexity. FastQC-0.11.8 also produces a report in HTML format. Then high-quality reads were aligned to the soybean genome (*Glycine max* Wm82.a4.v1) by STAR (Dobin et al., 2013). As transcripts per million (TPM) (Wagner et al., 2012) normalization is more consistent across technical replicates than other normalization methods. We normalized data using TPM for most of the downstream analysis (Li and Li, 2018), and log_2_ transformed raw read counts are used for quality control steps and AD-AE based confounders removal. Datasets with known batch effect information are corrected with the ComBat-Seq (Zhang et al., 2020). For the microarray data type, raw datasets are retrieved with GEOquey (Sean and Meltzer, 2007). After outliers were filtered out, we processed the CEL-type raw data with affy, an R package used to analyze oligonucleotide arrays and manufactured by Affymetrix (Gautier et al., 2004) and the oligo package developed by Carvalho and Irizarry (2010), which serves as a Bioconductor tool that supports R packages. Final data were normalized with GCRMA (Gharaibeh et al., 2008), which converts background-adjusted probe intensities to expression measures using the same normalization and summarization methods as the robust multiarray average (RMA) (Irizarry et al., 2003).

As shown in Supplementary Figure 1, 5,422 high-quality samples remained for further analysis, including 3,819 microarray samples and 1,603 RNA-seq samples. For each sample, we manually labeled its cultivar, tissue, development stage, and case-control information. All RNA-seq and microarray data analyzed in this work can be obtained from the European Nucleotide Archive^2^ and Gene Expression Omnibus (GEO), respectively.^3^ Accession numbers are summarized in Supplementary File 1.

### Removal of Confounders

As in Dincer et al. (2020), the AD-AE model consists of one standard autoencoder and an adversary network model that takes the embedded layer as input and predicts the confounders. Here, we used the data sources as confounder variates. The autoencoder network consists of an encoder network and a decoder network. The encoder network is defined as *f*_∅_:*X*→*Z*, which maps each sample *X*∈ℝ^*M*^ in the input layer to the embedding layer *Z*∈ℝ^D^, where M is the gene number of each sample. The decoder network tries to reconstruct X with the embedded layer Z. The optimize function is defined in Eqn. (1):


(1) $$m\,i\,n_{\Phi ,\psi }\,\text{E}\,{||x-g_{\psi }\,\left(f_{\Phi }\,\left(x\right)\right)||}_{2}^{2},$$

where Φ and ψ are the parameters for the encoder and decoder networks, respectively. In this study, after parameters optimization, for all tissue-specific expression data, the embedded layer size is set to 100, and the input layer sizes are the same as the gene number. We used one hidden layer for all the AE models, with the size of the half gene number, resulting in a 50%-dimension reduction. The minibatch size was set to 128, and we trained the model with the Adam optimizer using a learning rate of 0.0001. We applied the ReLU activation to all layers except the last layer, where we applied linear activation. The adversary model maps the embedding Æ to confounders. To reduce the confounding effects, after training, the autoencoder needed to converge to generate an embedding that contains less information about the confounder, and the adversary model needed to converge to reach a random prediction performance.

The adversary model *h_v_* is optimized with the following objective:


(2) $$m\,i\,n_{v}\,𝔼\,\left[L\,\left(h_{v}\,\left(x\right),c\right)\right]$$

where *c* is the confounder, and *L* is the loss function with categorical cross entropy loss. For the adversarial model, as in Dincer et al. (2020), a fully connected neural network has two hidden layers with 100 hidden nodes in each layer, and this network uses the ReLU activation function. The last layer’s number of nodes corresponds to the number of data sources, and this layer has softmax activation. First, we trained each model separately. We then used the following objective function for the alternative joint training.


(3) $$m\,i\,n_{\Phi ,\psi ,\nu }\,𝔼\,\left[{||x-g_{\psi }\,\left(f_{\Phi }\,\left(x\right)\right)||}_{2}^{2}-\lambda \,L\,\left(h_{v}\,\left(x\right),c\right)\right]$$

We first froze the weights of the adversary model and trained the autoencoder model for one epoch on a randomly selected minibatch of the data using stochastic gradient descent. We then froze the autoencoder model and trained the adversary model for an entire epoch to minimize Eqn. (2). For each dataset, we applied a fivefold cross validation to select the hyperparameters of autoencoder models, which is suitable considering the data set size. When training the model, we used 80% of the data for training with the rest left for validation, and we determined the optimal epochs based on the validation loss. We used the reconstructed data from the autoencoder model as input for our next step analysis. After removing confounders, for each tissue, data sets from different sources were more consistent and datasets from different tissues became more separate.

### Tissue-Specific Gene Identification, Gene Regulatory Networks, and Differential Network Construction

Instead of using the mean value across all samples as the gene expression value, we proposed using an autoencoder model to compress the gene expression vector to a lower dimension. The encoder network is defined as *f*_∅_:Y→*Z*, which maps each gene Y∈ℝ^N^ in the input layer to the embedded layer *Z*∈ℝ^D^, where N is the sample number of each gene. Here the input to the autoencoder model is the gene expression values across all samples. To get genes highly expressed in each tissue for each tissue-specific gene expression matrix, the embedding layer size was set to 1-dimension, the input layer size was set with the same sample number of each tissue. We used one hidden layer for all the AE models, with the size set as half the sample number, which resulted in a 50%-dimension reduction. The minibatch size was set to 12, and we trained the model with the Adam optimizer using a learning rate of 0.0001. We used the ReLU activation for all layers except the last layer, where we applied softplus activation. With the compressed gene expression matrix, we used τ as defined in Eqn. (4) to measure the tissue specificity of each gene.


(4) $$\tau =\frac{\sum_{i=1}^{n}\left(1-\hat{y_{i}}\right)}{n-1};\hat{y_{i}}=\frac{y_{i}}{\max_{1\leq i\leq n}\left(y_{i}\right)}$$

where *n* is the tissue number including the control condition. *y_i_* is the expression of the gene in tissue *i*. We used the 3rd quantile of all τvalues as the threshold value to filter highly expressed genes in each tissue and in the control condition.

For constructing tissue-specific GRNs, we used the same autoencoder model as that used to identify tissue-specific genes except that we set the embedding layer size to 64-dimension. However, the embedding layer size for the nodule tissue was set to size 32 (because only 47 samples exist for nodules). A total of 3,747 *Glycine max* known TFs were obtained and in combination with tissue-specific genes identified above, 662, 736, 781, and 617 TFs were highly expressed in leaf, root, seed, and nodule, respectively. Among these TFs, 110, 93, 155, and 110 are uniquely highly expressed in leaf, root, seed, and nodule, respectively. Some genes or TFs are not expressed in some tissues; therefore, they will not have predicted targets. Hence, the compressed expression matrix for each tissue is filtered to remove low variance genes by using the filterGenes function in the DGCA R package (Mckenzie et al., 2016; Zhou et al., 2020). GENIE3 ran under the default parameters setting and restricted the candidate regulators to the filtered tissue-specific TFs. By utilizing the embedded data sets, the filtered tissue-specific TFs and the GENIE3 (Huynh-Thu et al., 2010) algorithm, four tissue-specific (leaf, root, seed, and nodule) GRNs were constructed. To find TFs that play important roles in each GRN, we ranked all TFs based on their degree.

Understanding interactions that specifically exist in one tissue in comparison with those in the control conditions is important to the understanding of tissue-specific gene regulation. Therefore, we constructed the differential co-expression network of each tissue based on paired tissues and control expression data produced by the autoencoder model. Different from differential expression, differential co-expression operates on the level of gene pairs rather than individual genes. To deal with this, the Fisher z-transformation is employed as in Eqns. (5 and 6).


(5) $$z=\frac{1}{2}\,l\,o\,g_{e}\,\left(\frac{1+\rho }{1-\rho }\right)$$

(6) $$d\,z=\frac{\left(z_{1}-z_{2}\right)}{\sqrt{\left|s_{z_{1}}^{2}-s_{z_{2}}^{2}\right|}}$$

where ρ is the Pearson correlation coefficients, *z_1_* and *z_2_* corresponding to tissue-specific and control values, respectively, and $s_{z}^{2}$ refers to the variances of the z-score. Using the difference in z-scores, *dz*, a two-sided *p*-value can be calculated using the standard normal distribution and by adjusting the *p*-values for multiple hypotheses tests with the conservative Benjamini-Hochberg *p*-value adjustment method. Gene pairs can then be ranked based on the relative strengths of their differential correlation. To determine the top-ranked significantly changed hub genes, we sought to compare the differential correlation from paired tissues and the control genes co-expression network. To get hub genes, we calculated the average change in correlation for each gene with all others and set top-ranked genes as hub genes. This paper also presents a series of detailed tests to determine the difference in mean z-scores and adjust the *p*-value by random permutation samples 100 times. We used the DGCA (Mckenzie et al., 2016) R package to conduct the corresponding analyses in this study. DGCA offers a convenience function for extracting gene lists corresponding to the differential correlation, converting the resulting gene symbols to inputs for gene ontology enrichment testing and detecting functional modules. The whole pipeline is shown in Supplementary Figure 2.

## Results

### Tissue-Specific Gene Expression

By utilizing the autoencoder model, the collected soybean expression data were processed and their PCA plots were shown in Supplementary Figure 3. As shown in Supplementary Figure 3, after data reconstruction, tissue-specific expression signatures of data can still be maintained. By utilizing the autoencoder model and the τ index (between 0 and 1), genes with a τ index close to 1 were more specifically expressed in one tissue, while genes with a τ index closer to 0 are equally expressed across all tissues studied (Yanai et al., 2005).

**FIGURE 2:**
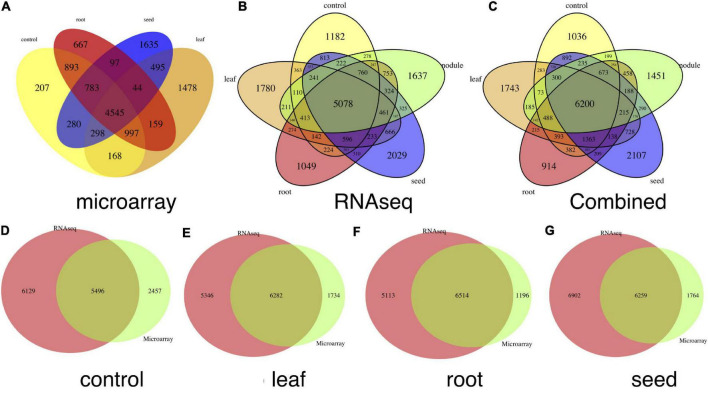
Venn plots of tissue-specific genes. **(A)** Number of tissue-specific genes in each tissue and control condition detected in the microarray data sets, **(B)** numbers of issue-specific genes in each tissue and control condition in the RNA-seq data sets, and **(C)** number of issue-specific genes in each tissue and control condition in combination with **(A,B)**. **(D–G)** Show common and tissue-specific genes detected in both microarray and RNA-seq data sets in control, leaf, root, and seed tissues, respectively.

**FIGURE 3:**
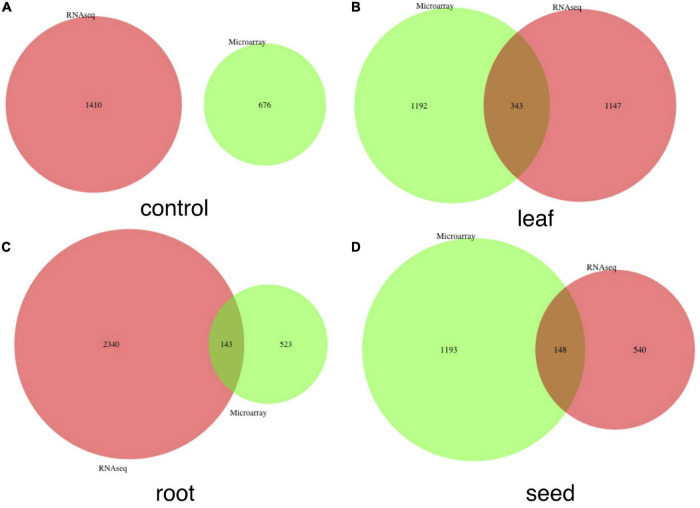
Venn plots of common genes detected in both microarray and RNA-seq data sets without the autoencoder model. These genes were detected directly with the τ index. **(A–D)** Correspond to numbers of common genes in the control, leaf, root, and seed, respectively.

We identified highly expressed genes in the leaf, root, seed, nodule and genes under the baseline condition, using both microarray and RNA-seq data sets. We combined the compressed 1-dimension expression value of each tissue and its control together and compared the global expression patterns among tissues to identify highly expressed tissue-specific genes. A gene is filtered as a tissue-specific gene only if it is highly expressed in that tissue compared to values of other tissues and the control mixture, which includes samples that do not involve exposure to the treatment or intervention from any studies (Supplementary Figures 4, 5). The Venn plot of combined tissue-specific genes in both microarray and RNA-seq data sets is shown in Figure 2C. In summary, we detected 1,743, 914, 2,107, and 1,451 genes highly expressed in leaf, root, seed, and nodule, respectively. The detailed gene list is shown in Supplementary File 2. Our method outperforms traditional methods in two ways: (1) with the autoencoder model, we can more accurately detect tissue-specific genes in each tissue, as shown in Figures 2A–G; (2) more common tissue-specific genes can be detected between the microarray and RNA-seq data types relative to traditional methods. The Venn plot of common tissue-specific genes between the microarray and RNA-seq identified using the traditional method is shown in Figure 3, which shows much fewer common tissue-specific genes between the microarray and RNA-seq data types.

To verify the accuracy of all the tissue-specific genes, we conducted a comprehensive literature search and finally collected 2,108, 1,908, 1,516, and 2,122 tissue-specific genes in total for leaf, root, seed, and nodule, respectively (Supplementary File 2), which are used as the benchmark datasets (Libault et al., 2009, 2010; Severin et al., 2010; Asakura et al., 2012; Jones and Vodkin, 2013; Brown and Hudson, 2015; Machado et al., 2020; Moisseyev et al., 2020). Supplementary Figure 6 shows the common genes between our results (denoted as O) and genes found in the eight benchmark studies (from A to H, with some detailed information from each study shown in Supplementary File 2). Many of these tissue-specific genes from the eight studies can always be detected by our method in contrast with other methods which usually have no gene in common, even with similar tissue-specific gene numbers for comparison. Different from other methods, we considered not only gene expression in specific tissues but also their expression under the control condition; hence, the genes detected by our methods are more likely highly expressed tissue-specific genes. To further verify the accuracy of our results, the top-10 ranked genes (genes with highest expression values in a tissue in comparison with other tissues and the baseline condition) of each tissue were searched in PubMed, as shown in Supplementary Table 1. Most of these top-ranked genes have direct PubMed publications that support their high expression in the corresponding tissue. A detailed tissue-specific expression of each gene can also be viewed through the link^4^ from the Soybean Expression Atlas. Considering the top-10 ranked nodule genes in Supplementary Table 1, we independently searched their average expression in various soybean tissues using the locus name (genome version: Gmax_275_Wm82.a2.v1), which confirmed their nodule-specific expression.

### Functional Annotation of the Top-Ranked Tissue-Specific Genes

To further validate the accuracy of the tissue-specific genes, we performed a GO functional enrichment analysis of the top 20 ranked tissue-specific genes using the TopGO package (Alexa et al., 2006). Only the top 20 significant GO terms of the unique genes are reported, except for nodules where 23 GO terms are reported. As shown in Supplementary File 2, many of the leaf-specific genes are those responding to red/blue light, such as Glyma.08G173700, Glyma.11G221000, Glyma.13G046200, Glyma.18G036400, Glyma.19G046600, and Glyma.19G046800. Among these genes, Glyma.11G221000 and Glyma.18G036400 also function in responding to cold and in the defense response to bacterial pathogens. Genes Glyma.13G046200, Glyma.19G046600, and Glyma.19G046800 also take part in the carbon fixation process. In a leaf, Glyma.14G061500 has a function in water transport and also in response to water deprivation. Another top-ranked gene is Glyma.13G347700, which is enriched in the processes of lateral root formation, responding to the abscisic acid, and it has a defense response to a bacterial pathogen. The Glyma.09G044200 gene takes part in processes of positive regulation of microtubule depolymerization, and it responds to cadmium ion and the regulation of multicellular organism growth (Berkowitz et al., 2008).

Many genes in seed are significantly enriched in lipid storage, embryo development, and seed germination processes (such as Glyma.10G246300 and Glyma.09G044200). Genes highly expressed in nodules are enriched in processes of nodulation and nitrogen fixation, which is consistent with previous knowledge (Elhady et al., 2020). Among the nodule-specific genes, Glyma.04G079200 responds to hypoxia and oxygen transport. Glyma.11G238800 participates in the pathogen defense response, and it is also highly expressed in the nodule tissue of *Lotus japonicus* (Guenther and Roberts, 2000). Glyma.10G199000 is highly expressed during nodule development (Marcker et al., 1984). Therefore, many of these tissue-specific genes have been experimentally verified, and others are good candidates for further exploration.

### Differential Network and Hub Genes

**FIGURE 4:**
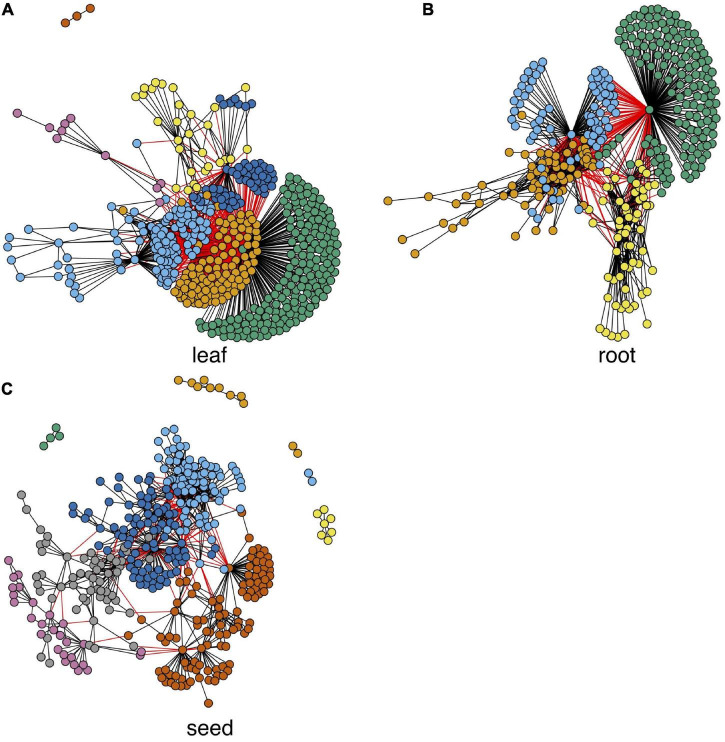
Tissue-specific differential networks and their functional modules. Each node represents a gene, and each edge represents a differential edge. Modules are marked by nodes in different colors.

An early study (Sonawane et al., 2017) shows that network edges have higher tissue specificity than network nodes. Therefore, comparing interaction changes between genes in a tissue and genes in the control group is important for understanding tissue-specific gene expression. We used the autoencoder compressed gene expression matrixes and the paired control gene expression matrix for tissue-specific differential network construction. Since genes with low average expression levels or low variance in expression levels are less likely to have biologically relevant differences between conditions, they were filtered out before further analysis as per previous protocol in studies (Mckenzie et al., 2016). We calculated the Pearson correlation value between any pair of genes in each tissue and also the corresponding value in the control condition; then, the significance of the difference between the two values was tested and the *p*-values were adjusted by the Benjamini-Hochberg method (Benjamini and Hochberg, 1995). We ranked the differential edges based on the adjusted *p*-values, and the top 1,000 ranked edges were selected for each tissue for the network construction. Results of each network after fast greedy clustering (Clauset et al., 2004) are shown in Figure 4, where there are 7, 4, and 10 modules in leaf, root, and seed differential networks, respectively. The nodule results are independently shown in the nodule section. We performed the biological process enrichment analysis for the modules in these tissues using the TopGO package (Alexa et al., 2006). Detailed gene module information and the GO annotation bubble plot are shown in Supplementary File 3.

To get hub genes in each network, we calculated each gene’s average gain or loss of correlation in the data set with all others (see details in “Materials and Methods” section). The top-ranked hub genes (genes with biggest gain or loss of correlation) of each network in Figure 4 are shown in Supplementary Table 2. Many of these genes in Supplementary Table 2 are known for their expression in the corresponding tissues. For example, Glyma.11G111400 is a fructose-bisphosphate aldolase protein, which is a key plant enzyme involved in glycolysis and the Calvin cycle in wheat and corn leaves (Lv et al., 2017). The root hub gene Glyma.07G248600 is a C2 domain-containing protein. Many C2 domain-containing proteins, such as CaSRC2-1, are known to be highly expressed in roots (Kim et al., 2008). The seed hub gene Glyma.13G295200 is a zinc finger CCCH domain-containing protein, which is known for its association with seed oil accumulation (Li et al., 2017).

### Tissue-Specific Gene Regulatory Networks and Transaction Factors

**FIGURE 5:**
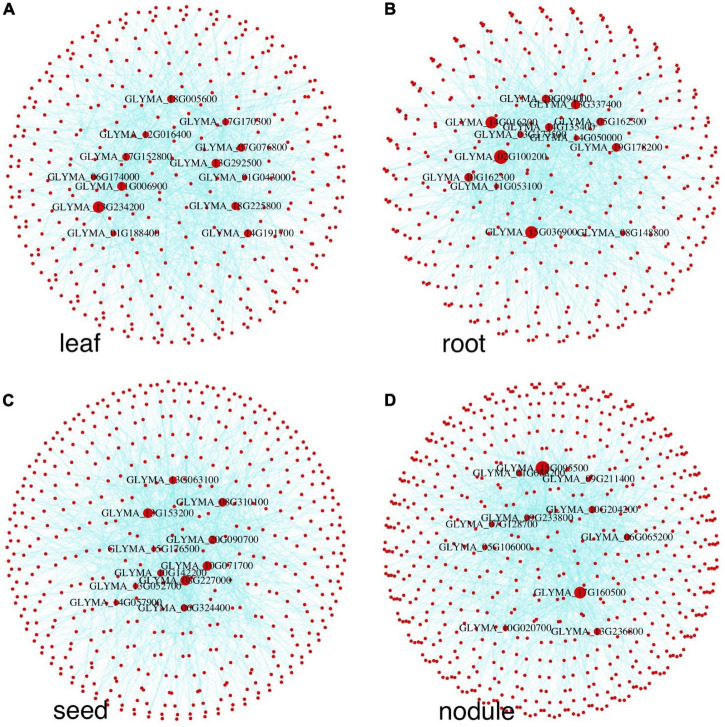
Tissue-specific GRNs of leaf, root, seed, and nodule. Red nodes represent genes, blue lines represent regulatory edges. Key regulatory genes are marked by the corresponding gene names. The node size is proportional to the node degree; thus, the bigger the node, the higher the node degree is.

GENIE3 (Huynh-Thu et al., 2010) was used for tissue-specific GRN construction (see the “Materials and Methods” section for details) after we compressed each tissue’s expression matrix to a low dimension. To find the optimal dimension size, we tuned our model using the DREAM 5 challenge *E. coli* expression data and the corresponding benchmark network with varying dimension sizes from 12 to 24, 32, 64, and 96, as well as using the original sample size. According to the area under the receiver operator characteristic (ROC) curves, the optimal dimension size was 64. For an expression matrix with more than 64 samples (leaf, root, seed, and control) we compressed them to the 64-dimension size. Other sample data (nodule) were compressed to the 32-dimension size due to having fewer samples than 64. For candidate regulators, we downloaded 3,747 soybean TFs from the PlantTFDB database (Jin et al., 2017). We combined the detected tissue-specific genes, 110, 93, 155, and 110, and from them, we found uniquely highly expressed TFs in leaf, root, seed, and nodule, respectively. Other parameters of GENIE3 were set as the default values. Prior to the analysis, we filtered out all the genes with low variance across samples as in Sun et al. (2020) by using the filterGenes function in the DGCA R package (Mckenzie et al., 2016). Because we only constructed tissue-specific GRNs, for each tissue only tissue-specific genes were considered, resulting in 660, 632, 737, and 582 genes left for leaf, root, seed, and nodule, respectively. For better visualization and comparison, each GRN was constrained to include only the top 1,000 high-weight edges calculated by GENIE3 (Walley et al., 2016; Huang et al., 2018). As shown in Figure 5, the top 5 central nodes in each network are all classified as biologically essential for the corresponding tissue.

The TF with the highest degree (number of connecting edges) in each GRN means it likely regulates many other genes or TFs, and such TFs are important in tissue-specific gene expression. We ranked all network nodes based on their degree. Supplementary Table 3 shows detailed information on the top-ranked TFs in the four tissues. Two root TFs are from the WRKY transcription factor family. Genes in the WRKY family play important roles in plants responding to microbial pathogens (Yang et al., 2017). The WRKY transcription factor genes are highly expressed in hairy roots and can enhance the resistance of soybean to the oomycete pathogen *Phytophthora sojae* (Cui et al., 2019). Another two root TFs are from the GRAS family, which broadly participates in many critical processes such as signal transductions, root radial elongations, axillary shoot meristem formations, and stress responses in plants (Bolle et al., 2000; Li et al., 2018). Overexpressing the GRAS family gene in hairy roots can improve the resistance of soybeans to drought and salt stresses (Wang et al., 2020). Three BZIP family TFs are top-ranked in seed. The BZIP genes play important roles in seed maturation and storage protein gene regulation (Lara et al., 2003; Wang Z. H. et al., 2019). The other two seed TFs are from the MIKC and MADS family, which often play potentially essential roles in seed development (Fan et al., 2013). Three nodule-specific TFs are from the C3H transcription factor family, one from NAC and one from the ERF family. The NAC family proteins are highly expressed during early symbiotic events in *Medicago truncatula* and *Sinorhizobium meliloti* (Lohar et al., 2007). The C3H and ERF family TFs are also important in the symbiosis process of *Lotus japonicus* (Asamizu et al., 2008).

### Nodule-Specific Genes and Gene Regulatory Network

**FIGURE 6:**
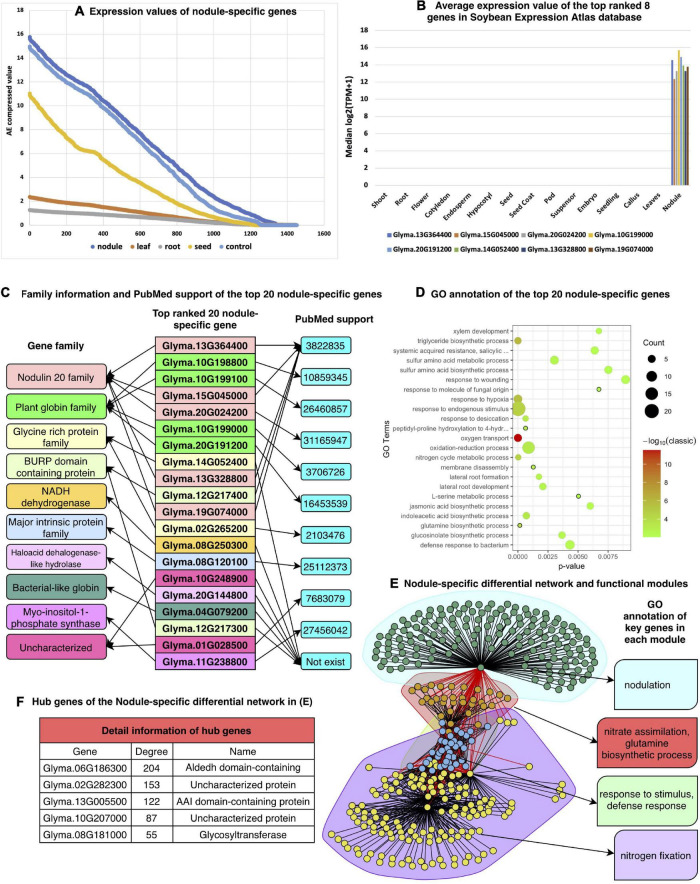
Results of nodule-specific gene analysis. **(A)** Expression values of 1,451 nodule-specific genes compared with those in other tissues, **(B)** validation of the expression patterns of top-ranked nodule-specific genes from the Soybean Expression Atlas database, **(C)** detailed gene family and PubMed support for the top-20 ranked nodule genes. Genes with the same color are in the same gene family. **(D)** GO biological process annotation of the top 20 nodule-specific genes, **(E)** differential correlation network of the nodule-specific genes and key function of each module, and **(F)** hub genes of each module.

The symbiosis between the rhizobium and soybean is a much cheaper and more effective agronomic practice for ensuring an adequate supply of nitrogen. Due to its agricultural importance, many efforts are underway to identify the underlying molecular mechanisms of the symbiosis process. As a result, many genes have been identified, such as the LysM receptor-like kinases, NFP and LYK3 (Radutoiu et al., 2003), which mediate the perception of Nod factors. SymRK is required for root nodule development (Chen et al., 2012) and miR393j-3p limits nodule development through repression of the nodulin gene ENOD93 (Yan et al., 2015). A higher percentage of NIN-like and C2H2 nodule-specific TFs has been reported (Libault et al., 2010; Severin et al., 2010). Furthermore, over 200 nodulins (organ-specific plant proteins induced during symbiotic nitrogen fixation) have been experimentally validated (Roy et al., 2020). However, considering the complexity of the symbiosis process, more genes are likely involved and need to be discovered. Figure 6 shows many nodule-specific highly expressed genes and their corresponding GRNs based on our method.

Figure 6A shows that all the detected nodule-specific genes are highly expressed only in nodules that compare with this type of gene in other tissues. Of the top 10 ranked nodule specific genes, eight also exist in the Soybean Expression Atlas database, except for the Glyma.10G198800 and Glyma.10G199100 genes. All eight nodule-specific genes are exclusively highly expressed in nodules and have no expression value in any other tissues, as shown in Figure 6B. Most of the top-ranked genes identified by our method match PubMed publications that support their high expression in the corresponding tissue as shown in Figure 6C. The bubble plot of the enriched biological processes of these genes is shown in Figure 6D.

According to the GO enrichment analysis results, many biological processes are related to the symbiosis process. For instance, copper is an essential nutrient for symbiotic nitrogen fixation, and cellular copper ion homeostasis is an important process in rhizobia-infected nodule cells (Senovilla et al., 2018). In *Lotus japonicus*, the expression of two thiamine biosynthesis genes, THI1 and THIC, is enhanced by inoculation with rhizobia but not by inoculation with arbuscular mycorrhizal fungi, and thiamine biosynthesis genes can promote nodule growth (Nagae et al., 2016). The ratios of sucrose/fructose in nodules can be changed in response to nitrate, indicating that nitrate affects sugar concentration in nodules (Streeter, 1981). Some genes are enriched in the process of responding to fructose, sucrose, etc. Other biological processes like the response to oxidative stress and response to hypoxia have all been shown to be involved in the soybean symbiosis process (Van Heerden et al., 2008; Zilli et al., 2011; Pucciariello et al., 2019).

As noted in the previous section, we constructed the nodule-specific differential regulatory network and found its functional modules. As shown in Figure 6E, four modules were detected, which are related to (1) nodulation, (2) nitrate assimilation as part of the glutamine biosynthetic process, (3) response to stimulus in a defense mode, and (4) nitrogen fixation, and all of them are well known for their relationship to the symbiosis process. Furthermore, except for the hub genes as in Figure 6F, many other genes are also related to the nodule function.

Of the top 100 ranked nodule-specific highly expressed genes, many symbiosis-related genes are identified, including four leghemoglobin gene Glyma.10G198800, Glyma.10G199100, Glyma.10G199000, and Glyma.20G191200, as well as nine nodulin genes (Glyma.13G364400, Glyma.15G045000, Glyma.20G024200, Glyma.13G328800, Glyma.19G074000, Glyma.06G216500, Glyma.02G204500, Glyma.17G073400, and Glyma.08G076800). Furthermore, some top-ranked TFs are also identified as symbiosis related. These include Glyma.01G159200 from the NIN-like family, Glyma.15G173300, Glyma.17G051400, and Glyma.09G014100 from the NF-YA/NF-YB family, seven genes (Glyma.13G094400, Glyma.05G106000, Glyma.06G303100, Glyma.12G100600, Glyma.15G069300, Glyma.17G065800, and Glyma.17G160500) from the MYB family and Glyma.01G101800, Glyma.02G144400, Glyma.14G110900, and Glyma.18G042300 from the C2H2 family (Supplementary File 2).

Many genes from these gene families are known to function in the symbiosis process. For example, a higher percentage of NIN-like and C2H2 nodule-specific TFs have been reported (Libault et al., 2010; Severin et al., 2010). Furthermore, NIN-like and C2H2 TFs are important in nitrate signaling (Konishi and Yanagisawa, 2013) and symbiosome differentiation during nodule development (Sinharoy et al., 2013). Therefore, our newly identified TFs are more likely to play important roles in the symbiosis process. We also found four nodule-specific ERF TFs (Glyma.03G112000, Glyma.07G114000, Glyma.09G072000, and Glyma.09G233800) that are essential for nodule differentiation and development (Vernie et al., 2008). Next, in the top 100 ranked nodule-specific highly expressed genes, we analyzed the 12 nodule-related module hub genes (Supplementary File 2) in the nodule-specific differential network. Notably, two of these genes (Glyma.18G041100 and Glyma.11G215500) are glutamine synthetase genes, which are tightly controlled enzymes located at the core of nitrogen metabolism. Glutamine synthetase catalyzes the first step in nitrogen assimilation, which is the ATP-dependent condensation of ammonium with glutamate (Seabra and Carvalho, 2015). The hub gene Glyma.17G045800 is a sucrose synthase gene, which has an essential function in the nitrogen fixation process (Gordon et al., 1999). Another hub gene, Glyma.08G181000, has been verified to function in the isoflavone biosynthetic pathway (Gupta et al., 2017). Due to the significant expression changes of these genes in the nodule tissue, they are likely to play important roles in the symbiosis process.

## Discussion and Conclusion

Transcriptome data is still the main data source for researchers to obtain useful information about plant biological processes and to identify key biomarker genes related to specific phenotypes. Each experimental study investigates gene expression in a range from a few samples to hundreds of samples. Due to the sample condition or method difference, even for the same plant tissue, different labs can obtain quite different results. Therefore, large-scale integration analysis of all the available datasets is needed to help us better understand gene expression from the systematic view. However, several factors make such analysis difficult; for instance, different studies utilize different platforms. The microarray platform is the most popular at first, but is dominated by the RNA-seq later. Besides, different studies use different kinds of plant samples under different treatments—or samples are collected at different development stages and time points. Over the years, several meta-studies have been conducted, but the scale and depth have been limited.

In this study, we systematically collected more than 7,000 raw sequencing data sets, which were mapped and processed in a uniform way. To our knowledge, this is the largest transcriptome analysis of soybeans until now. In the data normalization and processing, we proposed utilizing the unsupervised autoencoder model. Two features of unsupervised learning make it well suited to gene expression analysis. The first feature is the ability to train informative models without supervision, as it is challenging to obtain a high number of expression samples with coherent labels. Although many new expression profiles are released daily, the portion of the datasets with labels of interest is often too small. A second feature of unsupervised learning is: models are trained to extract patterns from the data without imposed hypotheses or restrictions. This aspect can be key to unlocking biological mechanisms unknown to the scientific community. To minimize the difference between data from different sources, we proposed using the unsupervised AD-AE machine learning model, which can efficiently remove confounders with the collected data sets. Because each tissue has many samples, to extract important signals from noises, the autoencoder model can efficiently compress expression values to a lower dimension. With the normalized and processed data sets, we analyzed highly expressed tissue-specific genes in leaf, root, seed and nodule. Besides, we constructed the tissue-specific GRNs and differential correlation networks based on these networks, and we identified key TFs, functional modules, and hub genes. According to our analysis, many identified genes have had the tissue-specific expression. The results were integrated into SoyKB. These tissue-specific genes may help researchers test hypotheses in downstream experiments and functional genomics studies. However, several limitations exist in this study. Although many tissues and development stages were involved in the collected datasets, here we only showed results for seed, root, leaf, and, nodules as these four tissues occupied the most samples. More attention will be paid to other tissue analysis in the future.

## Data Availability Statement

The original contributions presented in the study are included in the article/Supplementary Material, further inquiries can be directed to the corresponding author/s.

## Author Contributions

LTS, DX, TJ, and GS contributed to the conception and design of this study. LTS, CX, SZ, and LS processed the datasets. LTS, CX, and LS performed the data analyses. LTS wrote the first draft of the manuscript. All authors contributed to manuscript revision and also read and approved the submitted version.

## Conflict of Interest

The authors declare that the research was conducted in the absence of any commercial or financial relationships that could be construed as a potential conflict of interest.

## Publisher’s Note

All claims expressed in this article are solely those of the authors and do not necessarily represent those of their affiliated organizations, or those of the publisher, the editors and the reviewers. Any product that may be evaluated in this article, or claim that may be made by its manufacturer, is not guaranteed or endorsed by the publisher.

## Footnotes

1
https://github.com/FelixKrueger/TrimGalore

2
https://www.ebi.ac.uk

3
https://www.ncbi.nlm.nih.gov/geo/

4
http://venanciogroup.uenf.br/cgi-bin/gmax_atlas/index.cgi

## Supplementary Material

Supplementary Figure 1 Sample number distribution. **(A)** Sample number distribution of all the collected samples, **(B)** sample number of each tissue of RNAseq datasets, and **(C)** sample number of each tissue represented by microarray datasets.

Supplementary Figure 2 Detailed pipeline of tissue-specific gene identification, GRNs, and differential network construction.

Supplementary Figure 3 PCA plots of the AD-AE reconstructed datasets. **(A)** The PCA plot of the reconstructed microarray control dataset, **(B)** the PCA plot of the reconstructed RNA-seq control dataset, **(C)** the PCA plot of the reconstructed microarray case dataset, with samples from leaf, root, and seed, and **(D)** the PCA plot of the RNA-seq case dataset, with samples from leaf, root, seed, and nodule. Each color represents a different tissue, and the same shape represents samples from the same tissue.

Supplementary Figure 4 Expression heatmap of highly expressed tissue-specific genes detected in the microarray data sets. **(A–D)** Correspond to tissue-specific genes in control, leaf, root, and seed, respectively.

Supplementary Figure 5 Expression heatmap of highly expressed tissue-specific genes detected in the RNA-seq data sets. **(A–E)** Correspond to tissue-specific genes in control, leaf, root, seed, and nodule, respectively.

Supplementary Figure 6 (1) Tissue-specific genes collected from eight benchmark studies **(A–H)** and results from our method and (2) heatmap of common gene numbers between results from different studies. O represents our own results. Leaf_O represents leaf genes detected by our method and leaf_A represents leaf genes detected in study **(A)**. Others are correspondingly defined in the figure.

Supplementary File 1 Detailed information of all samples used in this study. Microarray_Control shows all microarray samples in control condition. Microarray_Case shows all microarray samples with various treatments. RNA-seq_Control shows all RNA-seq samples in the control condition. RNA-seq_Case shows all RNA-seq samples with various treatments. RNA-seq SRAruninfo shows all the SRA IDs in the RNA-seq raw sequencing data that we used. Microarray GEOid shows all the GEO IDs in the microarray raw sequencing data that we used. In-house sample data show all the sample information provided by our collaborators.

Supplementary File 2 Detailed results of all tissue-specific genes.

Supplementary File 3 Gene and GO annotation information of all the functional modules in Figure 5.
