# Supplementary material for: Large-Scale Integrative Analysis of Soybean Transcriptome Using an Unsupervised Autoencoder Model
Source: Front Plant Sci. 2022 Mar 3;13:831204. doi: 10.3389/fpls.2022.831204 (PMC8927983; doi:10.3389/fpls.2022.831204)
Supplement: Supplementary file 1 [file Table_1.DOCX]

Supplementary Materials

# Supplementary Tables

**Table S1.** PubMed support of the top-ranked 10 gene in each tissue

| leaf gene id | name | PMID |
| --- | --- | --- |
| Glyma.13G046200 | ribulose-bisphosphate carboxylase small chain (rbcS) | 26214418 |
| Glyma.11G221000 | 26S proteasome regulatory complex, ATPase RPT4 | 32072682 |
| Glyma.18G036400 | RCA gene | 29882786 |
| Glyma.19G046800 | Ribulose bisphosphate carboxylase small chain | 12228356 |
| Glyma.04G167900 | light-harvesting chlorophyll-protein complex | 33050902 |
| Glyma.19G046600 | ribulose-bisphosphate carboxylase small chain (rbcS) | 26569351 |
| Glyma.08G173700 | Photosystem II 10 kDa polypeptide, chloroplastic | 32072682 |
| Glyma.05G128000 | light-harvesting complex II chlorophyll a/b binding protein 1 (LHCB1) | 30665369 |
| Glyma.19G106800 | glyceraldehyde-3-phosphate dehydrogenase (NDP+) (phosphorylating) (GAPA) | 26214418 |
| Glyma.08G082900 | light-harvesting complex II chlorophyll a/b binding protein 1 (LHCB1) | 30665369 |
| root gene id | name | PMID |
| Glyma.09G092700 | Repetitive proline-rich cell wall protein 1 | Not exist |
| Glyma.17G186600 | Elongation factor 1-alpha | 27806110 |
| Glyma.08G230500 | Pathogenesis-related protein Bet v I family (Bet_v_1) | 31960590 |
| Glyma.01G083300 | Aspergillus nuclease S(1) / Single-stranded-nucleate endonuclease | 29329437 |
| Glyma.09G023000 | Peroxidase | 26689712 |
| Glyma.08G125800 | Receptor-like serine/threonine-protein kinase | 32152894 |
| Glyma.12G073100 | L-ascorbate peroxidase | 25268626 |
| Glyma.05G114900 | Elongation factor 1-alpha | 26706070 |
| Glyma.17G030200 | G.max mRNA from stress-induced gene (H4) | 28066473 |
| Glyma.06G186300 | Aldedh domain-containing protein | 28444301 |
| seed gene id | name | PMID |
| Glyma.10G207100 | Cysteine proteinase | 30519252 |
| Glyma.09G044200 | TCTP domain-containing protein | 29570733 |
| Glyma.13G363300 | Seed maturation protein PM30 | 30113693 |
| Glyma.17G186600 | elongation factor 1-alpha (EEF1A) | Not exist |
| Glyma.10G246300 | Beta-conglycinin alpha’ subunit | 31892538 |
| Glyma.09G185500 | Dehydrin | 31596499 |
| Glyma.03G144400 | seed maturation protein | 31869408 |
| Glyma.14G061500 | Aquaporins | 30634702 |
| Glyma.12G095700 | Seed maturation protein PM37 | Not exist |
| Glyma.05G198000 | Knot1 domain-containing protein | 23555009 |
| nodule gene id | name | PMID |
| Glyma.13G364400 | Nodulin | 3822835 |
| Glyma.10G198800 | Leghemoglobin C3 | 10859345 |
| Glyma.10G199100 | Leghemoglobin A | 26460857 |
| Glyma.15G045000 | Nodulin-22 | 3822835 |
| Glyma.20G024200 | Nodulin-C51 | 31165947 |
| Glyma.10G199000 | Leghemoglobin C1 | 3706726 |
| Glyma.20G191200 | Leghemoglobin C2 | 16453539 |
| Glyma.14G052400 | Glycine rich protein family | Not exist |
| Glyma.13G328800 | Nodulin | 3822835 |
| Glyma.19G074000 | Nodulin | 3822835 |

**Table S2.** Hub genes in tissue-specific differential network

| leaf hub gene | degree | names |
| --- | --- | --- |
| Glyma.11G111400 | 432 | Fructose-bisphosphate aldolase |
| Glyma.10G145900 | 228 | Uncharacterized protein |
| Glyma.05G052400 | 88 | 40S ribosomal protein S13 |
| Glyma.06G165100 | 80 | AAA_12 domain-containing protein |
| Glyma.10G261200 | 65 | Peptidase of plants and bacteria (BSP) |
| root hub genes | degree | names |
| Glyma.14G048900 | 228 | Fe2OG dioxygenase domain-containing protein |
| Glyma.07G248600 | 118 | C2 domain-containing protein |
| Glyma.05G052400 | 68 | 40S ribosomal protein S13 |
| Glyma.19G007200 | 61 | Cinnmoyl coa reductase-like protein |
| Glyma.06G036700 | 49 | Phytocyanin domain-containing protein |
| seed hub genes | degree | names |
| Glyma.13G295200 | 85 | Zinc finger ccch domain-containing protein 2-related |
| Glyma.05G180300 | 55 | Ethylene insensitive 3-like 4 protein-related |
| Glyma.18G166600 | 46 | Phosphatidylinositol glycan, class S (PIGS) |
| Glyma.20G213900 | 39 | SHSP domain-containing protein |
| Glyma.05G142500 | 37 | MFS domain-containing protein |
| nodule hub genes | degree | names |
| Glyma.06G186300 | 204 | Aldedh domain-containing protein |
| Glyma.02G282300 | 153 | Uncharacterized protein |
| Glyma.13G005500 | 122 | AAI domain-containing protein |
| Glyma.10G207000 | 87 | Uncharacterized protein |
| Glyma.08G181000 | 55 | Glycosyltransferase |

**Table S3.** Top-ranked TFs in each tissue and their family information.

| leaf gene id | degree | name | family |
| --- | --- | --- | --- |
| Glyma.13G234200 | 68 | Auxin response factor | ARF |
| Glyma.11G006900 | 53 | CRC domain-containing protein | CPP |
| Glyma.13G292500 | 53 | TCP domain-containing protein | TCP |
| Glyma.07G076800 | 49 | Homeobox-leucine zipper protein (HD-ZIP) | HD-ZIP |
| Glyma.14G191700 | 48 | Dnj (hsp40) homolog, subfamily A, member 3A | MYB |
| Glyma.18G005600 | 48 | SBP-type domain-containing protein | SBP |
| Glyma.18G225800 | 48 | Homeobox-leucine zipper protein (HD-ZIP) | HD-ZIP |
| Glyma.17G152800 | 46 | Two-component response regulator-like, APRR2 | ARR-B |
| Glyma.17G170300 | 41 | Sf18 - floral homeotic protein apetala 2 | AP2 |
| Glyma.12G016400 | 39 | Nucleoporin-like protein 2 (NUPL2, CG1) | C3H |
| seed gene id | degree | name | family |
| Glyma.08G227000 | 60 | BZIP domain-containing protein | bZIP |
| Glyma.13G153200 | 58 | BZIP domain-containing protein | bZIP |
| Glyma.10G071700 | 56 | BZIP domain-containing protein | bZIP |
| Glyma.08G310100 | 51 | Agamous-like mads-box protein agl1-related | MIKC_MADS |
| Glyma.20G090700 | 50 | MYB/HD-like transcription factor | MYB |
| Glyma.13G063100 | 47 | Mini zinc finger protein 2 | ZF-HD |
| Glyma.06G324400 | 43 | MADS domain transporter AGL11 | MIKC_MADS |
| Glyma.10G142200 | 42 | MYB/HD-like transcription factor | MYB |
| Glyma.13G052700 | 34 | Mads box protein | MIKC_MADS |
| Glyma.14G057900 | 33 | Ethylene-responsive transcription factor abi4 | ERF |
| root gene id | degree | name | family |
| Glyma.02G100200 | 84 | NAC domain-containing protein | NAC |
| Glyma.15G036900 | 74 | GRAS domain-containing protein | GRAS |
| Glyma.14G016200 | 73 | Wrky transcription factor 20-related | WRKY |
| Glyma.13G337400 | 55 | GRAS domain-containing protein | GRAS |
| Glyma.10G162300 | 52 | Helix-loop-helix DN-binding domain (HLH) | bHLH |
| Glyma.19G178200 | 52 | AP2-like factor, euAP2 lineage (AP2) | AP2 |
| Glyma.19G094000 | 51 | Squamosa promoter-binding-like protein 13a-related | SBP |
| Glyma.05G162300 | 49 | HSF_DOMAIN domain-containing protein | HSF |
| Glyma.14G135400 | 48 | Wrky transcription factor 11-related | WRKY |
| Glyma.03G177500 | 39 | AP2-like factor, euAP2 lineage (AP2) | AP2 |
| nodule gene id | degree | name | family |
| Glyma.15G095500 | 84 | Zinc finger ccch domain-containing protein 34-related | C3H |
| Glyma.17G160500 | 71 | MYB transcription factor | MYB_related |
| Glyma.10G204200 | 44 | MYB/HD-like transcription factor | G2-like |
| Glyma.13G236800 | 43 | C3H1-type domain-containing protein | C3H |
| Glyma.06G065200 | 41 | Uncharacterized protein | TALE |
| Glyma.01G088200 | 36 | NAC domain-containing protein | NAC |
| Glyma.07G128700 | 35 | Effector of transcription2-related | HRT-like |
| Glyma.09G233800 | 35 | AP2/ERF domain-containing protein | ERF |
| Glyma.10G020700 | 34 | Zinc finger ccch domain-containing protein 32 | C3H |
| Glyma.05G106000 | 31 | MYB transcription factor | MYB_related |
