# Supplementary figures and images for: Large-Scale Integrative Analysis of Soybean Transcriptome Using an Unsupervised Autoencoder Model
Source: Front Plant Sci. 2022 Mar 3;13:831204. doi: 10.3389/fpls.2022.831204 (PMC8927983; doi:10.3389/fpls.2022.831204)

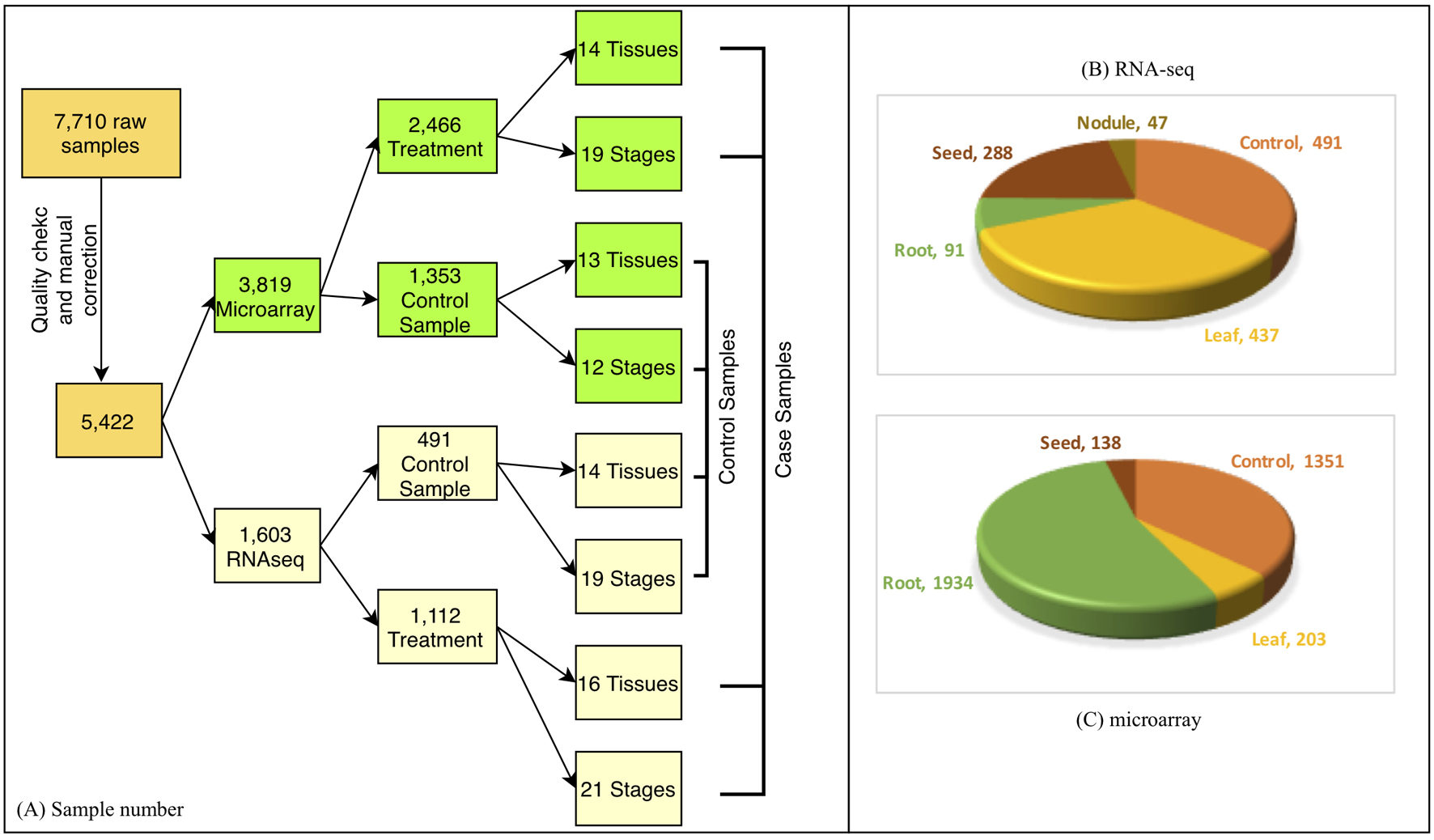

Supplement: Supplementary Figure 1 — Sample number distribution. (A) Sample number distribution of all the collected samples, (B) sample number of each tissue of RNAseq datasets, and (C) sample number of each tissue represented by microarray datasets. [file Image_1.JPEG]

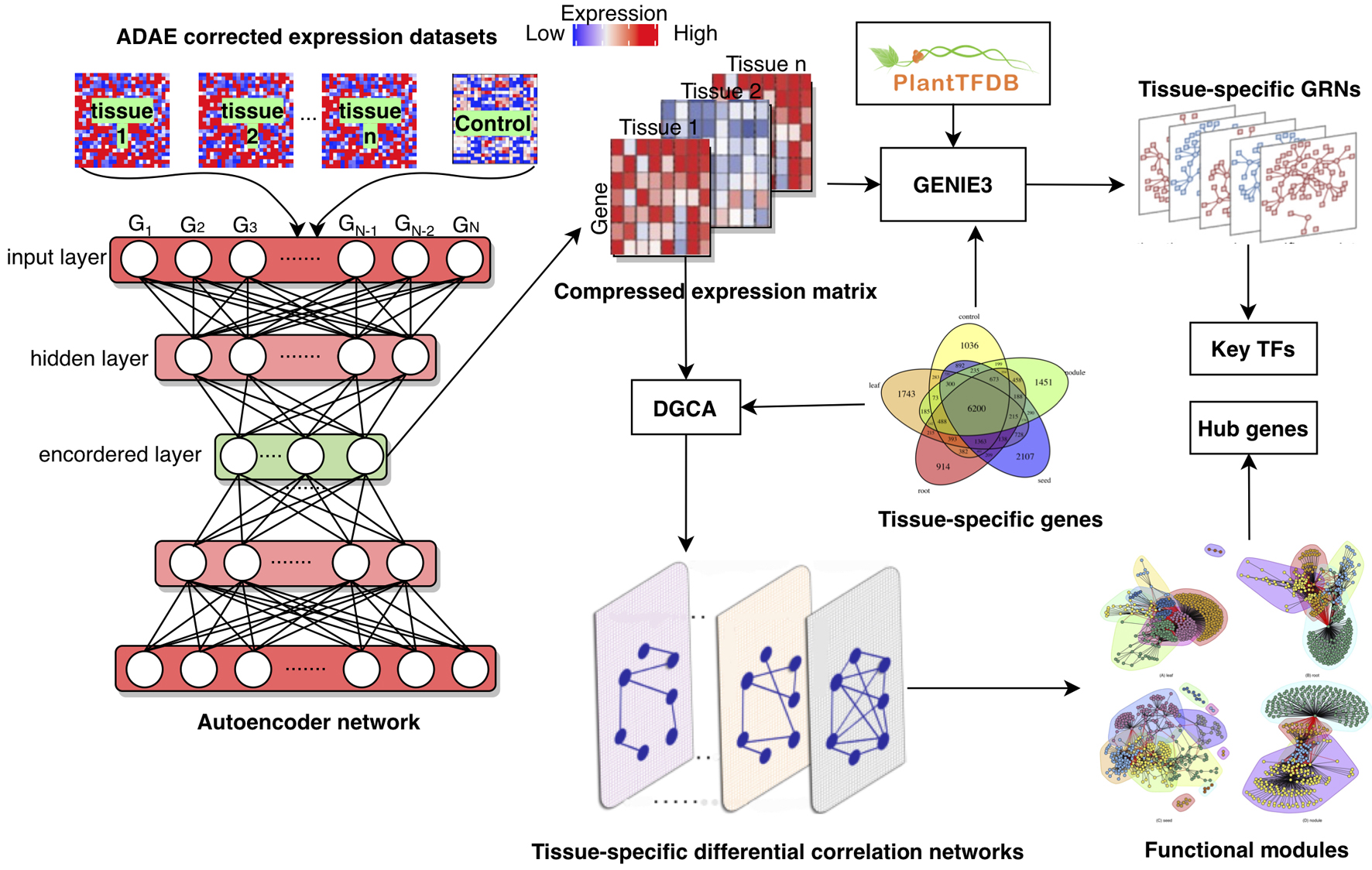

Supplement: Supplementary Figure 2 — Detailed pipeline of tissue-specific gene identification, GRNs, and differential network construction. [file Image_2.JPEG]

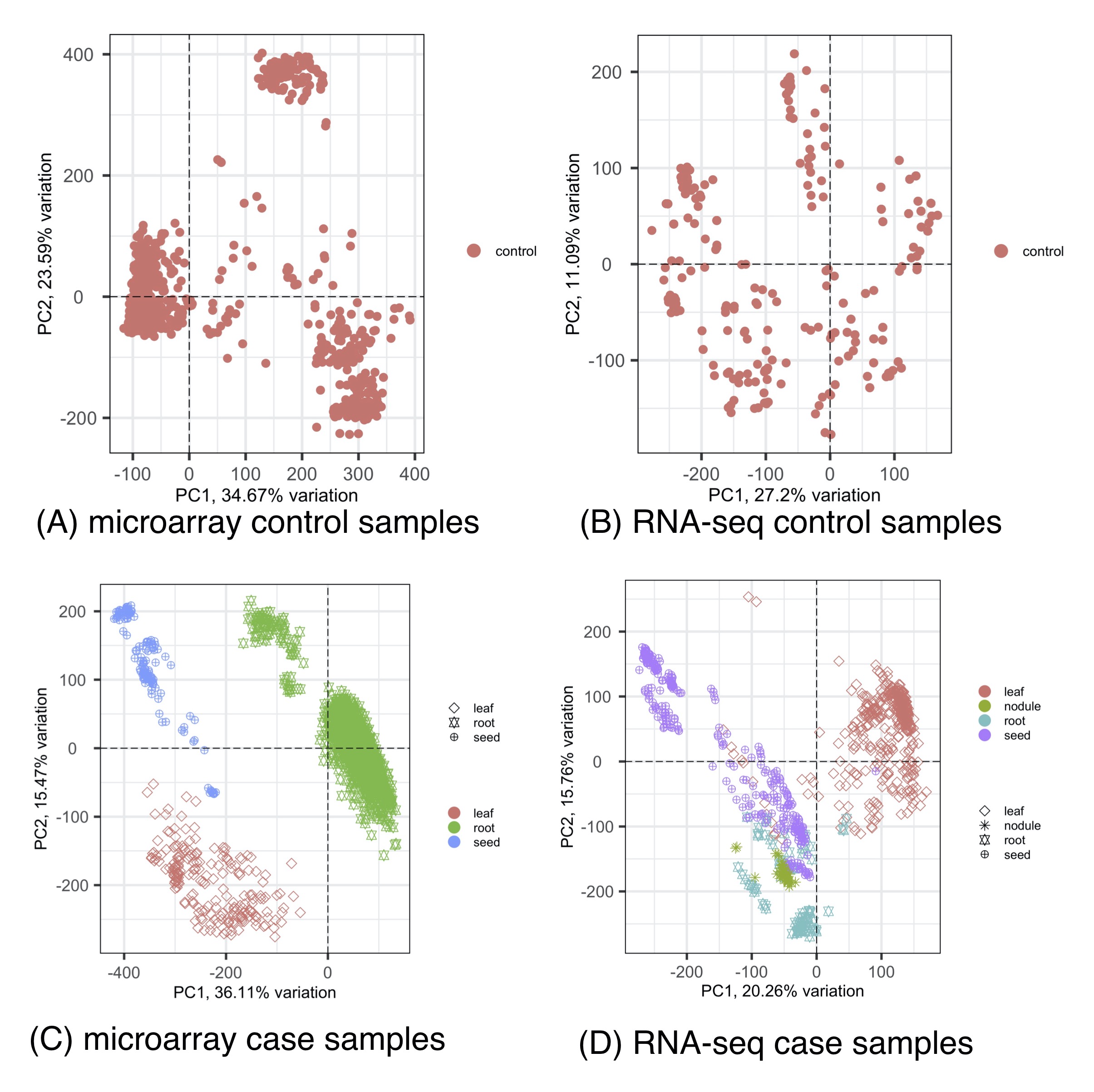

Supplement: Supplementary Figure 3 — PCA plots of the AD-AE reconstructed datasets. (A) The PCA plot of the reconstructed microarray control dataset, (B) the PCA plot of the reconstructed RNA-seq control dataset, (C) the PCA plot of the reconstructed microarray case dataset, with samples from leaf, root, and seed, and (D) the PCA plot of the RNA-seq case dataset, with samples from leaf, root, seed, and nodule. Each color represents a different tissue, and the same shape represents samples from the same tissue. [file Image_3.JPEG]

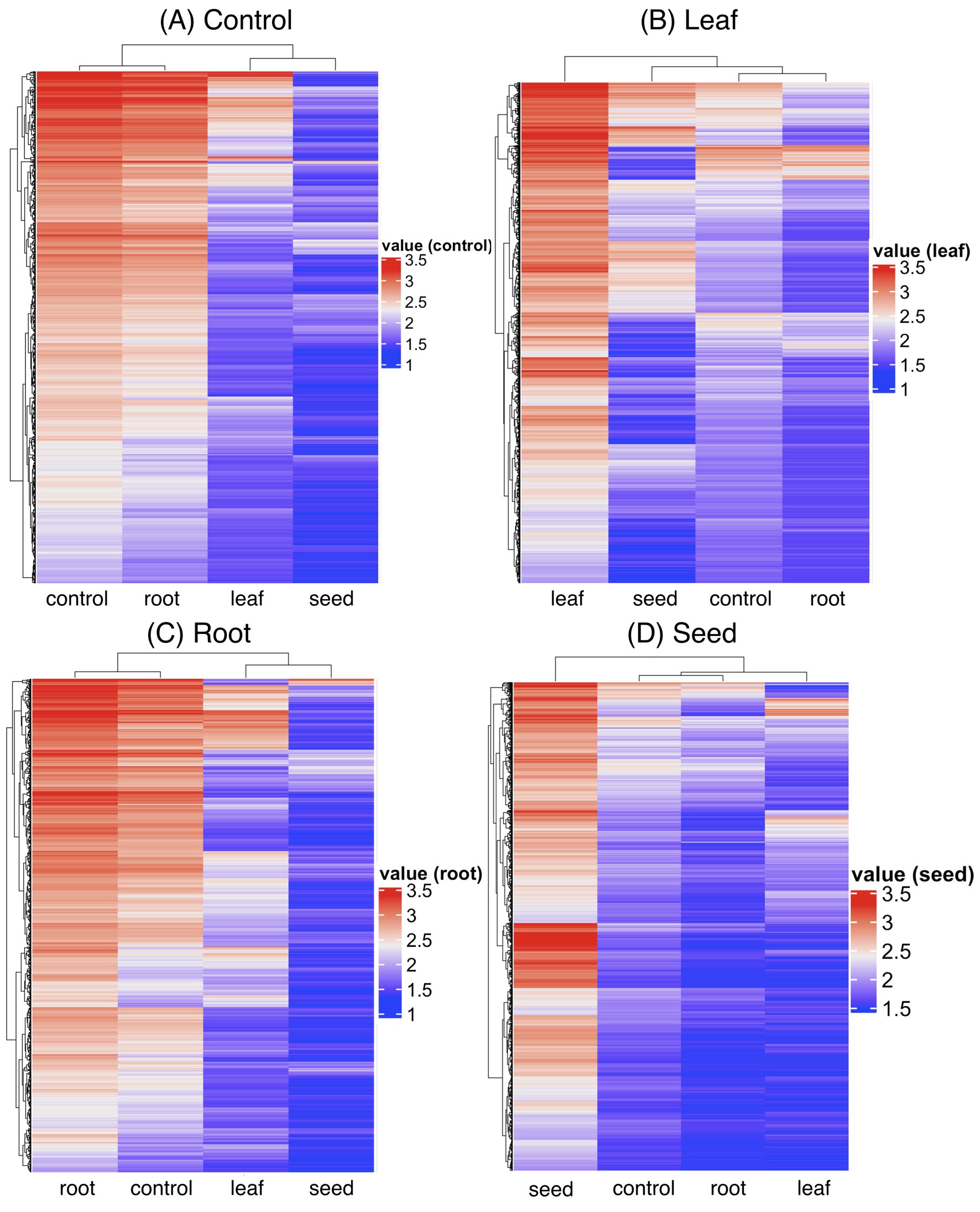

Supplement: Supplementary Figure 4 — Expression heatmap of highly expressed tissue-specific genes detected in the microarray data sets. (A–D) Correspond to tissue-specific genes in control, leaf, root, and seed, respectively. [file Image_4.JPEG]

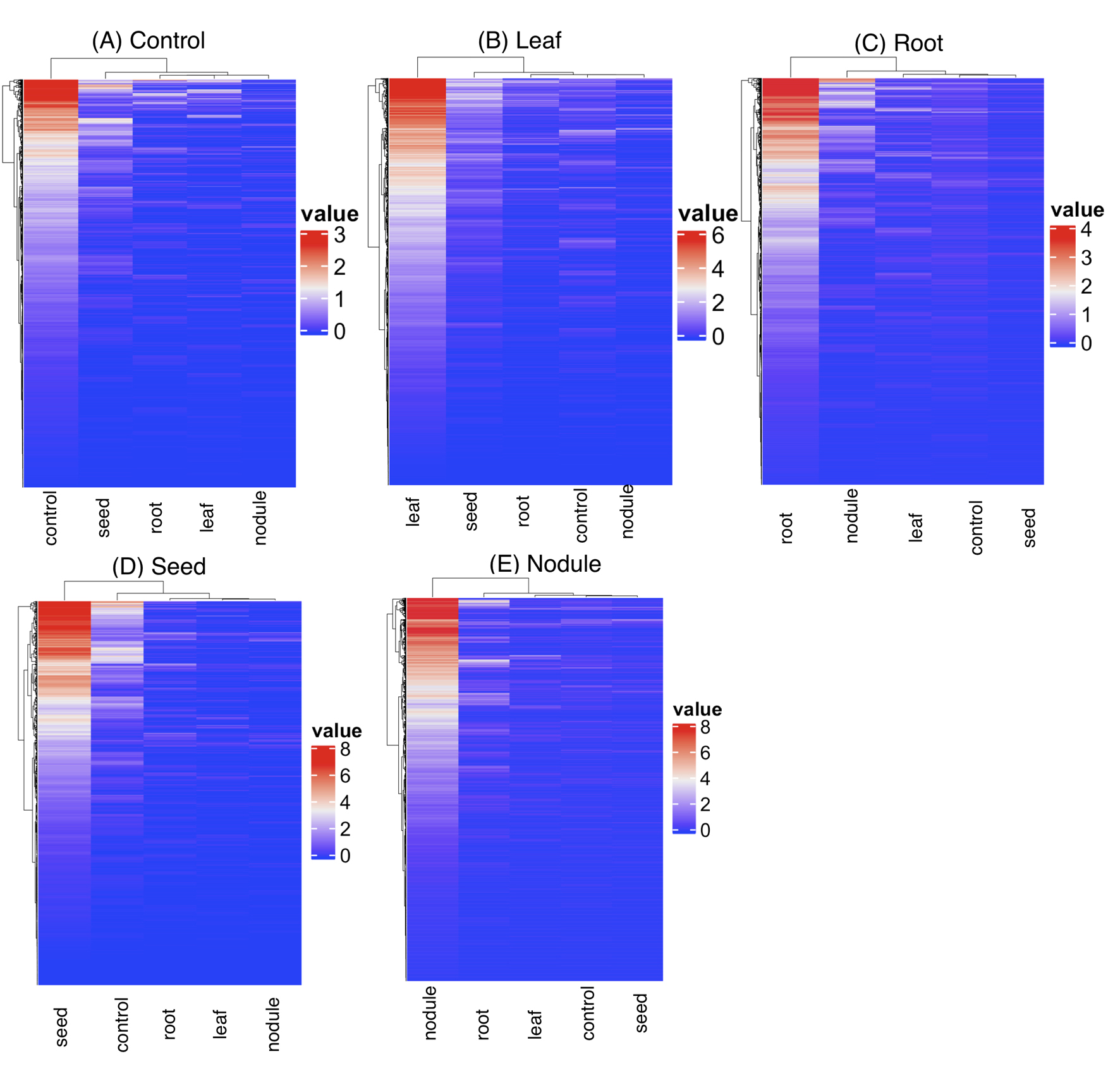

Supplement: Supplementary Figure 5 — Expression heatmap of highly expressed tissue-specific genes detected in the RNA-seq data sets. (A–E) Correspond to tissue-specific genes in control, leaf, root, seed, and nodule, respectively. [file Image_5.JPEG]

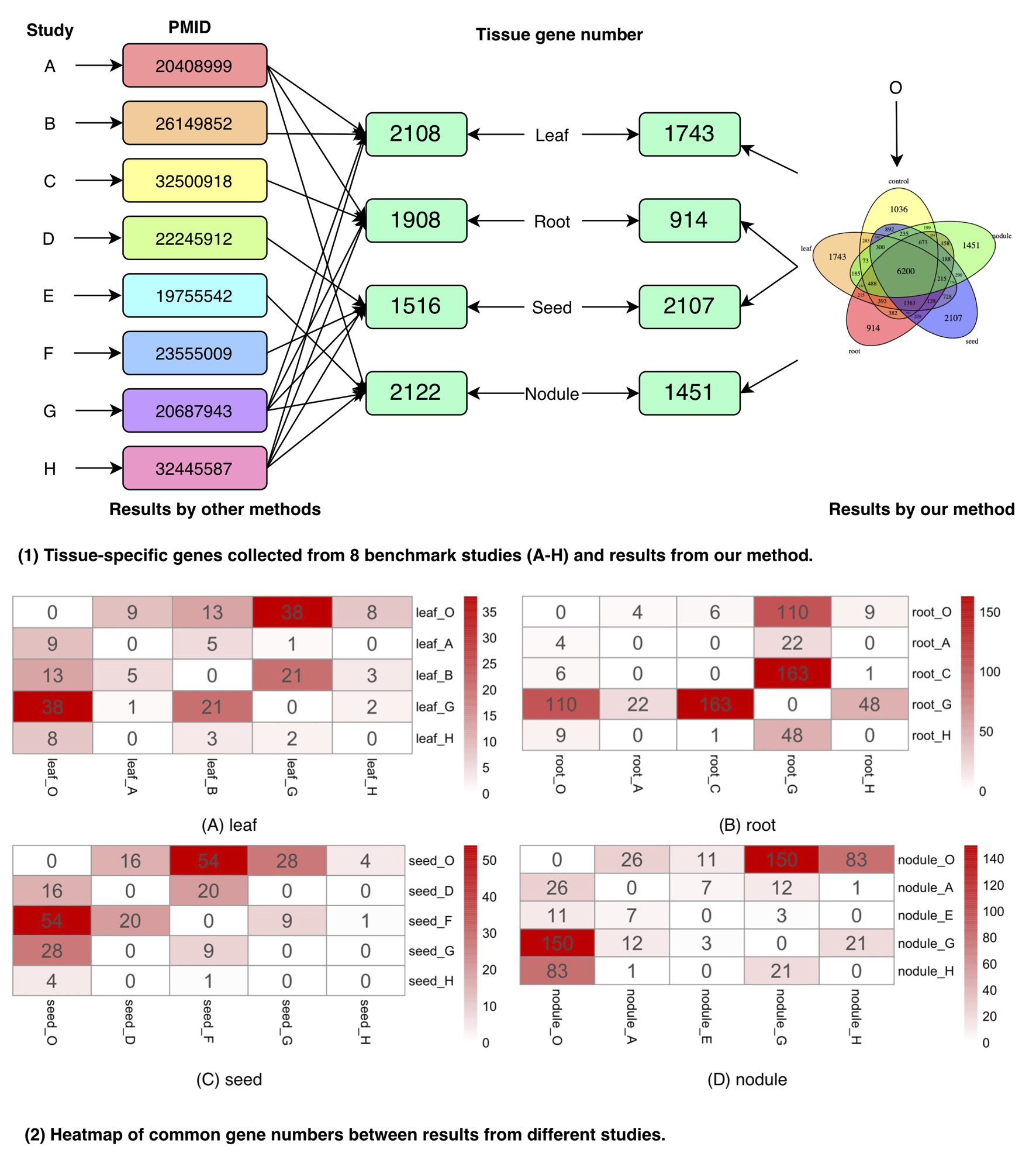

Supplement: Supplementary Figure 6 — (1) Tissue-specific genes collected from eight benchmark studies (A–H) and results from our method and (2) heatmap of common gene numbers between results from different studies. O represents our own results. Leaf_O represents leaf genes detected by our method and leaf_A represents leaf genes detected in study (A). Others are correspondingly defined in the figure. [file Image_6.JPEG]
